# The emerging phenotype of nonalcoholic fatty liver disease in lean individuals: what’s different?

**DOI:** 10.3389/fendo.2025.1693123

**Published:** 2025-10-20

**Authors:** Priyankar Dey

**Affiliations:** Department of Biotechnology, Thapar Institute of Engineering and Technology, Patiala, Punjab, India

**Keywords:** lean NAFLD, lean NASH, visceral adiposity, sarcopenia, PNPLA3, fibrosis, all-cause mortality, gut microbiota

## Abstract

**Background:**

Non-alcoholic fatty liver disease (NAFLD), currently referred to as metabolic dysfunction-associated steatotic liver disease (MASLD), occurring in adults of normal weight, represents a unique emerging phenotype apart from obesity-related NAFLD. Notwithstanding a normal body mass index (BMI), this phenotype poses considerable metabolic and hepatic risk, undermining conventional obesity-focused paradigms of fatty liver disease.

**Methods:**

This comprehensive review integrates global epidemiological data, molecular investigations, and clinical research to elucidate the distinct pathogenesis, risk factors, natural history, and treatment of lean NAFLD. Essential bibliographical databases were screened for research on disease prevalence, genetic determinants, metabolic characteristics, and long-term consequences.

**Results:**

Lean NAFLD impacts 5-20% of the worldwide NAFLD population, with a greater frequency in Asian cohorts (~45%). It is characterized by visceral obesity, sarcopenia, and significant genetic determinants (variants of PNPLA3, TM6SF2, and MBOAT7) in normal BMI individuals. Gut dysbiosis and modified bile acid metabolism further delineate its pathophysiology. Importantly, lean NAFLD presents similar or elevated risks for all-cause mortality (1.6-fold increase), advanced fibrosis, cirrhosis, hepatocellular carcinoma, and cardiovascular disease compared to obese NAFLD, despite a lower prevalence of metabolic comorbidities.

**Conclusion:**

Lean NAFLD is a clinically relevant condition necessitating customized diagnostic and therapeutic approaches. Lifestyle modifications focusing on moderate weight reduction (3-5%), fructose and cholesterol restrictions, and resistance exercise are highlighted. Future investigations should emphasize consistent classifications, non-invasive biomarkers, and medicines tailored to lean NAFLD phenotypes.

## 1 Introduction

Nonalcoholic fatty liver disease (NAFLD) encompasses a spectrum of hepatic conditions characterized by an excess accumulation of triglycerides in hepatocytes, exceeding 5% liver fat, in the absence of significant alcohol use or other secondary etiologies of liver disease (1). This categorization requires the careful elimination of other etiologies, including viral hepatitis, autoimmune hepatitis, genetic disorders, and drug-induced liver injury (2). The acceptable limit for alcohol consumption to prevent NAFLD is generally considered below 20 g/day for males and below 10 g/day for women; however these thresholds may vary and are not uniformly defined (3). The disease spectrum ranges from simple hepatic steatosis (NAFLD), which often shows a benign course with little risk of progression, to nonalcoholic steatohepatitis (NASH). NASH is a more severe form characterized by hepatic steatosis, inflammation, hepatocellular ballooning, and possibly varying degrees of fibrosis (4). The progression from NAFLD to NASH is critical, since NASH markedly increases the risk of severe conditions, including advanced fibrosis, cirrhosis, hepatocellular carcinoma (HCC), and liver failure.

Recently, a significant shift in nomenclature has occurred, with NAFLD being often referred to as Metabolic Dysfunction-Associated Steatotic Liver Disease (MASLD) (5). This move indicates a deeper understanding of the disease pathogenesis, evolving from a purely excluding diagnostic criteria (e.g., non-alcoholic) to one that acknowledges the strong metabolic factors regardless of the body mass index (BMI). This re-framing emphasizes that metabolic dysfunction is essential to disease characterization and etiology (6). Employing this updated nomenclature connects the discussion with contemporary medical discourse, demonstrating an advanced understanding of the subject. This also offers a basis for a detailed analysis of metabolic variables in lean individuals, since the core definition of MASLD now includes metabolic dysregulation as a key diagnostic requirement (7).

Historically, metabolic liver disease has been primarily linked to obesity, with most afflicted individuals classified as overweight or obese (8). This robust correlation has often resulted in the presumption that fatty liver disease is mostly a result of significant weight gain and its associated metabolic disorders. A significant and increasingly acknowledged percentage of patients diagnosed with NAFLD and NASH are of normal weight, characterized by a standard BMI (9). This phenotype, referred to as ‘lean NAFLD’ or ‘lean NASH,’ questions the prevailing notion that hepatic fat deposition and inflammation are exclusively associated with obesity. The classification of lean NAFLD as a unique phenotype underscores a significant deficiency in conventional diagnostic and screening frameworks. Due to the absence of obesity, a typical clinical indicator of steatosis, these patients often remain unrecognized and underreported (10, 11). The lack of conventional obesity-related risk factors often results in a postponed diagnosis of liver steatosis or injury. Individuals with lean NAFLD often exhibit no symptoms, with the condition being identified accidentally during standard blood tests or imaging examinations (12). The delay in diagnosis might result in severe outcomes, as the disease may proceed to more severe stages prior to the commencement of therapeutic care (11). Despite a lean stature, these individuals are sometimes labeled as ‘metabolically obese’ owing to underlying metabolic dysregulation that BMI alone does not adequately reflect (13). This highlights that metabolic health is not only indicated by body weight, and that a healthy BMI does not exclude the possibility of substantial underlying metabolic dysfunction that might lead to liver disease. The increasing acknowledgment of lean NAFLD requires an expanded diagnostic perspective and a more sophisticated comprehension of the intricate interactions among genetic, metabolic, and environmental variables that lead to fatty liver disease, transcending a sole emphasis on obesity.

## 2 Definition and diagnostic criteria of lean NAFLD/NASH

### 2.1 BMI cut-offs and ethnic considerations

The notion of ‘lean NAFLD’ is mostly determined by the BMI thresholds, which differ markedly between ethnic groups. In individuals of non-Asian heritage, lean NAFLD is often diagnosed in those with NAFLD and a BMI under 25 kg/m² (14). Conversely, for persons of Asian descent, a lower BMI threshold of ≤23 kg/m² is advised to define lean NAFLD (15). These ethnic-specific thresholds are essential, as they recognize that many cultures may encounter metabolic risk and develop fatty liver disease at lower BMI levels than others. This distinction is a clear suggestion from the Centers for Disease Control and Prevention (CDC) and the World Health Organization (WHO), who acknowledge that metabolic risk may present at varying BMI thresholds across various groups. (16). It is also essential to differentiate lean NAFLD from non-obese NAFLD, despite the sometimes-interchangeable usage of both terms. Non-obese NAFLD encompasses a wider classification that includes individuals deemed overweight (BMI ranging from 25-29.9 kg/m² for non-Asians, or 23-27.5 kg/m² for Asians), as well as those who are of lean physique (14). This however, particularly examines the lean phenotype, characterized by a normal-range BMI based on race-specific thresholds (16). The inconsistency in BMI thresholds and the differentiation between lean and non-obese NAFLD in diverse studies (11) provide a considerable obstacle in epidemiological investigation and clinical equivalence. The absence of a universally standardized definition can result in variability in reported prevalence rates and complicate the interpretation of research findings, highlighting the persistent necessity for more consistent diagnostic criteria for research purposes to enhance data comparability across studies.

### 2.2 Exclusion criteria and diagnostic challenges

The diagnosis of NAFLD, especially for the lean phenotype, relies on a meticulous procedure of exclusion. It requires the complete exclusion of substantial alcohol intake, but specific thresholds may differ (1). A thorough assessment is necessary to exclude other recognized etiologies of hepatic steatosis or chronic liver disease beyond alcohol use. This encompasses, but is not restricted to, viral hepatitis (e.g., Hepatitis B and C), autoimmune hepatitis, genetic disorders such as hemochromatosis, Wilson’s disease, and α1-antitrypsin deficiency, in addition to drug-induced liver injury (e.g., methotrexate, amiodarone, tamoxifen, corticosteroids, etc.) (2). Other less prevalent but significant etiologies, including celiac disease, thyroid disorders, lipodystrophy, and familial hypobetalipoproteinemia, should also be considered (17). Specifically, excess fat is typically considered physiologically detrimental, but evidence suggests it may provide protection against overnutrition by acting as a buffer against metabolic risk factors (18). In the case of lipodystrophy, the lack of adipose tissue results in the transfer of fat to skeletal muscle and the liver, ending in a metabolic syndrome characterized by severe insulin resistance (19). In certain individuals, leptin insufficiency causes overnutrition, resulting in significant ectopic fat buildup and severe metabolic syndrome. In non-obese NASH, leptin levels are elevated or comparable to controls, in contrast to the decreased levels noted in lipodystrophy; adiponectin levels are significantly diminished in the majority of studies (20).

A notable diagnostic issue in lean NAFLD arises from the lack of obesity, a prevalent clinical marker for steatosis. Indeed, obesity-targeted interventions, such as stringent calorie restriction, may be unsuitable and perhaps detrimental for patients with lean NAFLD. Excessive weight reduction can aggravate sarcopenia and dietary deficits while neglecting critical aspects such as visceral adiposity and hereditary influences. Management could prioritize moderate weight reduction, nutritional quality, and resistance training to maintain muscle mass. The absence of prominent obesity often results in the issue being neglected or inadequately acknowledged by healthcare professionals (11). Individuals with lean NAFLD are generally asymptomatic, with the condition often identified accidentally via standard medical assessments, such as blood tests indicating abnormal liver enzymes or imaging investigations demonstrating hepatic steatosis (2). The asymptomatic characteristic, together with the lack of conventional risk factors, leads to a delayed diagnosis of liver steatosis or injury, thereby individuals may have more advanced liver disease upon diagnosis (11). This situation underscores the urgent necessity for enhanced clinical observation and modified screening strategies for lean populations, as existing screening frameworks, which predominantly depend on BMI as a key metric, may unintentionally overlook a significant number of individuals who are silently advancing towards severe liver disease.

### 2.3 Non-invasive diagnostic tools and limitations

The conclusive diagnosis of NAFLD often depends on the presence of fatty infiltration identified using imaging techniques such as ultrasonography, magnetic resonance imaging (MRI), or histological examination of liver biopsy (1). A liver biopsy is the definitive method for accurately diagnosing NASH, since it enables the histological evaluation of inflammation, hepatocyte ballooning, and fibrosis, distinguishing NASH from simple steatosis (21). However, liver biopsy is an invasive technique that entails risks, expenses, and patient discomfort, making it unfeasible for extensive screening or regular monitoring (22). As a result, non-invasive tests (NITs) have been developed and are being progressively used for fibrosis staging and risk assessment in patients with NAFLD. These include serum-based indices, including the Fibrosis-4 (FIB-4) index and the NAFLD Fibrosis Score (NFS), with imaging modalities such as transient elastography (TE, e.g., FibroScan) and magnetic resonance elastography (MRE) (22). Additionally, in recent days, the evolving significance of Mac-2 binding protein glycosylation isomer (M2BPGi) and growth differentiation factor 15 (GDF15) as biomarkers for metabolic liver disease has been recognized. M2BPGi functioning as a non-invasive indicator for liver fibrosis and disease advancement, whereas GDF15 serves as a crucial modulator of metabolic homeostasis, insulin sensitivity, and mitochondrial stress (23–25), collectively providing complementary perspectives for diagnosis, risk assessment, and therapeutic oversight. The NITs may assist in identifying subjects at elevated risk of advanced fibrosis or cirrhosis, thus minimizing the need for liver biopsies and directing referral paths to experts. They may be conducted upon diagnosis and thereafter repeated at intervals ranging from 6 months to 2-y, dependent upon the degree of fibrosis and the response to treatments (16). Sequential testing, including the integration of a serologic test with an imaging test, may enhance diagnostic precision and reduce uncertain outcomes (16).

Notwithstanding their usefulness, existing NITs has limitations, especially with lean NAFLD. Ultrasound, while economical and readily accessible, has inadequate accuracy for hepatic steatosis below 30%, often underreporting the actual frequency of NAFLD, particularly moderate steatosis, which may represent a substantial fraction in lean individuals (26). Moreover, NITs for conclusive NASH diagnosis remain costly and have not been well established for extensive clinical use (27). The dependence on invasive liver biopsy for conclusive NASH diagnosis, together with the constraints of NITs, constitutes a substantial obstacle to precise epidemiological evaluation and the early detection of lean NASH. This circumstance suggests that existing prevalence statistics for lean NASH may be underestimated, highlighting an urgent need for additional reliable, accessible, and validated non-invasive biomarkers and imaging methods, particularly designed for this phenotype, to enable early and exact diagnosis.

## 3 Global prevalence and epidemiology of lean NASH

**Figure 1:**
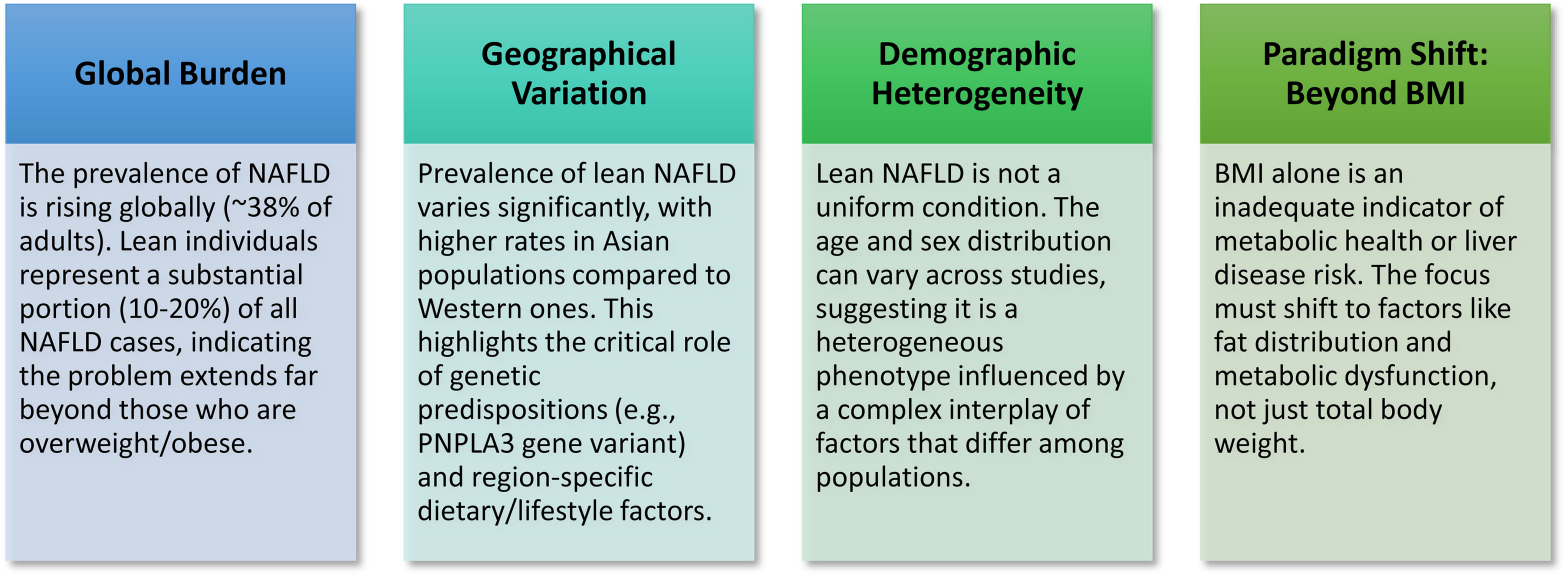
Key concepts in lean NAFLD/NASH: Lean NAFLD/NASH is a significant and growing subtype of fatty liver disease that occurs in individuals with a normal Body Mass Index (BMI), challenging the conventional obesity-centric view of the condition.

A distinct phenotype exists in lean NAFLD/NASH condition in terms of global burden, geographical variation, demographic heterogeneity, and metabolic features (**Figure 1**).

### 3.1 Overall prevalence of NAFLD and lean NAFLD

The worldwide incidence of NAFLD is significant and is on the increase, indicating an escalating public health issue. Recent estimations indicate that the prevalence of NAFLD in adults varies from 23% to 38% (13). Recent meta-analyses reveal a more than 50% rise in the prevalence of NAFLD over the last 30-y, with rates escalating from around 25% during 1990–2006 to 38% in 2016-2019 (28). In the growing NAFLD population, lean individuals represent a notable and more acknowledged segment. Estimates indicate that around 10% to 20% of all NAFLD patients are of normal weight (11). Meta-analyses indicate that the prevalence of lean NAFLD is estimated at 19.2% among the overall NAFLD population and 5.1% in the general population (14). NASH, the more advanced variant of NAFLD, has a worldwide frequency estimated at 5-7% among the general population (28). Nonetheless, this statistic significantly escalates among certain high-risk populations, such as persons with type 2 diabetes, the estimated incidence of NASH exceeds 7-folds, approximating 37% (28). Utilizing data from 1040 NAFLD patients over a decade, another research group demonstrated that lean Indian NAFLD patients have fewer metabolic risk variables yet comparable liver disease severity to their non-lean counterparts with NAFLD (29). Specifically, it was demonstrated that the lean patients had markedly reduced incidences of central obesity, hypertension, and metabolic syndrome compared to overweight patients. However, no notable differences were observed in steatosis, fibrosis markers, or biopsy-confirmed NASH or advanced fibrosis severity. Collectively, the escalating incidence of NAFLD, together with the significant and growing percentage of afflicted lean individuals, signifies an expanding condition that likely exceeds the conventional obesity-focused perspective. This indicates that public health measures aimed only at weight reduction may be inadequate to mitigate the total burden of NAFLD/NASH. A more sophisticated strategy is necessary, one that considers the many underlying risk factors present in lean populations, transcending a sole emphasis on BMI as the principal risk indicator.

### 3.2 Geographical variations and ethnic predispositions

The frequency of lean NAFLD varies significantly among geographical locations and ethnic groups, underscoring the complex interaction of environmental and genetic variables in its expression (30) (**Table 1**). Asian population, notably, has a greater frequency of lean NAFLD than the Western counterpart (38). Research demonstrates that the prevalence of lean NAFLD in Asian populations varies significantly from 5-45%, whereas in European people, it generally ranges from 5-20% (38). A study in rural India indicated a lean NAFLD prevalence of 5.1% among adults with a BMI <23 kg/m², with a significant 75% of all NAFLD cases occurring in those with a BMI below 25 kg/m² (26). A comprehensive research in Korea revealed a NAFLD prevalence of 12.6% among non-obese individuals (BMI <25 kg/m²), which escalated to 16% when only examining lean individuals (BMI <23 kg/m²) (26). Research conducted in China has shown lean NAFLD prevalence rates of 7.27% in non-obese adults and 18% in those with a BMI <24 kg/m². Conversely, the Dionysos study conducted in northern Italy indicated a lean NAFLD prevalence of 16.4% (26).

**Table 1:** Global prevalence and characteristics of lean NAFLD/NASH.

Category	Population/Region/Country	Prevalence of NAFLD	Prevalence within NAFLD population	Prevalence in general population)	BMI cut-offs for Lean NAFLD	Additional findings	Reference
Global	General population	30.05% (95% CI: 27.88 to 32.32%)	25.3%	11.2%	<25 (Western population), <23 (Asian population)	Increased liver-related mortality compared to obese NAFLD; robust genetic associations (e.g., PNPLA3)	(31, 32)
Global	Non-obese NAFLD	–	40.80%	12.10%	<25 (all regions)	39% with NASH; 29.2% with significant fibrosis; increased cardiovascular-related mortality	(14)
Global (Projected)	2040 Forecast	55.70%	–	–	–	Rapid escalation in female and non-metabolic syndrome cohorts	(33)
Asia	General population	29.6–31.6%	19.2–25.3%	5.1–7.9%	<23 kg/m²	Lean MASLD associated with more severe fibrosis; driven by GGT/ALP (not BMI/metabolic factors)	(14, 31, 34–36)
Western Countries	MAFLD cohort	36.83%	8.03%	2.83%	<25 kg/m²	Similar metabolic burden to obese MAFLD; 3.56-times more liver-related mortality	(35)
Latin America	General population	44.37% (highest globally)	–	–	<25	Urbanization contributing to rapid increase; inadequate lean-patient data	(32, 34)
Middle East & North Africa	General population	36.53%	–	–	<25	Genetic/epigenetic factors contributing to the high lean prevalence	(32)
North America	General population	31.20%	12%	0.6/100,000	<25	30-times higher risk of acute coronary syndrome; relatively common in Asian-Americans and older females	(37)
Europe	General population	25.10–26.9%	~50% (non-obese NAFLD)	–	<25	Highest proportion of non-obese NAFLD; high liver-related mortality in lean patients	(14, 34, 35)
Africa	General population	13.5% (globally lowest)	–	–	<25	Lack of comprehensive data; NAFLD burden may increase due to increased obesity	(34)

This significant regional and ethnic heterogeneity is not accidental. It seems to be closely associated with distinct dietary patterns and lifestyle characteristics characteristic of various geographical regions. Diets rich in fructose, low in protein and dietary fiber, along with sedentary lifestyles and inadequate sleep, have been linked to the frequency of lean NAFLD in some geographical regions (13). Moreover, genetic predispositions significantly contribute to elucidating these ethnic inequalities (39). Certain genetic variations, particularly those in PNPLA3, are more common in Asian populations and independently increase vulnerability to NAFLD, regardless of weight (39). The significant regional and ethnic disparities in lean NAFLD prevalence, especially its elevated rates among Asian people, indicate that a universal approach to understanding and addressing NAFLD is inadequate. This discovery highlights a crucial interaction among distinct genetic origins, conventional food habits, and changing lifestyles across various areas. Thus, it requires the formulation of culturally sensitive and ethnically customized public health treatments and research methodologies to successfully tackle the worldwide prevalence of lean NAFLD.

### 3.3 Demographic factors: age and sex distribution

The demographic characteristics of lean NAFLD patients, especially for age and sex distribution, exhibit a complicated and even contradicting landscape across several studies. Numerous studies show that lean NAFLD is often encountered in elderly adults, with some findings indicating a greater prevalence among females (13). Recent data from the Global NAFLD/NASH Registry indicated that lean NASH patients were often older and more frequently of Asian descent (16). Nonetheless, several studies provide contradictory results about both age and gender distribution. Certain research indicates that lean NAFLD patients may be under 40-y of age, whilst others emphasize that they may be above 60-y of age (40). Some research shows a greater incidence of lean NAFLD in men (40), whilst others suggest a higher frequency in females (13). Some individuals see no substantial disparities in incidence between men and females (40). A research, stratified by age and sex, indicated that men under 50-y have a heightened tendency for acquiring the lean NAFLD phenotype, however, no significant sex differences were seen after the age of 50-y (40). A research comparing lean and obese NAFLD groups revealed a higher prevalence of men in both categories, with a more pronounced male predominance in the obese group (41).

The discrepancies in demographic characteristics, especially regarding age and gender, among many studies indicate that lean NAFLD is not a homogeneous condition (42). Rather, it seems to be a heterogeneous phenotype shaped by a complex interaction of elements that may present differently across diverse cohorts and communities. This heterogeneity highlights the need of comprehensive patient phenotyping in research and clinical practice to identify distinct subgroups that may benefit from tailored diagnostic and treatment strategies. Comprehending these subtleties is essential for advancing toward a more individualized strategy for NAFLD treatment.

### 3.4 Associated risk factors in lean NASH

Individuals with lean NAFLD/NASH, while possessing a normal body mass index, are not metabolically healthy and have several underlying risk factors that contribute to the condition. These characteristics often vary in their significance or particular expression relative to those seen in obese NAFLD (**Table 2**).

**Table 2:** Key risk factors and clinical characteristics of lean NAFLD/NASH (2, 11, 13).

Risk factor/characteristic	Description in lean NAFLD/NASH	Comparison to obese NAFLD/NASH	Clinical implication
Visceral Adiposity	Elevated central/intra-abdominal adiposity despite a normal body mass index. Frequently surpasses the visceral fat area of certain obese individuals	A greater amount of visceral adiposity; obese individuals possess more peripheral and truncal fat. Adipocytes in lean individuals may exhibit significant malfunction	The Body Mass Index (BMI) is inadequate; prioritize the assessment of fat distribution. Visceral fat is a significant contributor to disease progression.
Insulin Resistance (IR)	Often involved in lipolysis and *de novo* lipogenesis. Frequently characterized as “metabolically obese.”	Less prevalent or less severe than in obese NAFLD, however still markedly elevated compared to healthy lean controls.	Even mild insulin resistance can induce disease; early identification of subclinical metabolic risk factors is essential.
Dietary Habits	Elevated fructose, elevated cholesterol, diminished protein, and diminished fiber consumption. Inactive lifestyle, poor sleep	The quality and composition of the food are more crucial than the total caloric surplus	Targeted dietary interventions (fructose/cholesterol restriction) are vital, even without aggressive weight loss.
Genetic Susceptibility	Significant involvement; variations such as PNPLA3 (rs738409), TM6SF2 (rs58542926), MBOAT7 (rs72613567), and ApoB are essential.	Frequently independent of obesity/insulin resistance; increased incidence of certain variations in lean non-alcoholic fatty liver disease.	Genetic screening can identify individuals at risk; future medicines may be tailored to certain genes.
Sarcopenia	Frequent correlation; gradual decline in muscle mass and integrity. Bidirectional association with NAFLD.	The prevalence markedly rose in NASH patients irrespective of BMI	Muscle health directly influences liver dysfunction; therapies aimed at muscle mass and function represent innovative therapeutic strategies
Differential Metabolites	Unique profiles; modified bile acid metabolism (elevated total bile acids), increased serum uric acid, and particular lipid patterns	May have elevated HDL and reduced TG/ALT compared to non-lean individuals, however inferior to healthy lean controls.	Prospective non-invasive biomarkers for diagnosis and monitoring; new therapeutic targets
Altered Gut Microbiota	Unique profile; decreased Firmicutes, elevated LPS-producing Gram-negative bacteria (Chinese); modified Ruminococcaceae, Dorea (Caucasian).	Distinct from obese NAFLD; particular bacterial alterations observed.	The gut-liver axis is significant; microbiome-targeted therapies such as probiotics and dietary modifications may represent distinctive therapeutic approaches
Age	Commonly observed in adults beyond 40 years of age. There are contradictory findings.	Generally older than overweight or obese NAFLD patients in several studies	Heterogeneous phenotype necessitates comprehensive patient phenotyping
Sex	Inconsistent results; certain research indicate males, others females, while some reveal no disparity	Results differ each study; males under 50 may exhibit a heightened probability	Heterogeneous phenotype necessitates comprehensive patient phenotyping.
Metabolic Syndrome Components	Dyslipidemia, hypertension, and type 2 diabetes mellitus are present, albeit less commonly than in obese non-alcoholic fatty liver disease.	Reduced prevalence of T2DM, dyslipidemia, hypertension, metabolic syndrome, cardiovascular disease, and cirrhosis in comparison to obese NAFLD.	Even minor metabolic disturbances can induce NAFLD; early identification of subclinical risk factors is crucial.

#### 3.4.1 Visceral adiposity and ‘metabolically obese normal weight’

A distinguishing feature of lean NAFLD patients is the presence of elevated central adiposity, particularly visceral fat, despite a normal or reduced BMI (43). This contrasts with obese or overweight individuals who often have more peripheral and truncal subcutaneous adiposity (44). Visceral fat exhibits more metabolic activity and has a stronger correlation with metabolic syndrome compared to subcutaneous fat (45). In some non-obese individuals, the visceral fat area may surpass that of obese individuals, highlighting its major significance as a contributing factor to disease development (13). A recent study shows that central obesity is an independent factor influencing advanced fibrosis in lean NAFLD patients (46). This study investigating 170 lean NAFLD patients identified central obesity in 56.5% patients. Patients with central obesity were mostly females, were diagnosed with hypertriglyceridemia and metabolic syndrome, aggravated liver steatosis identified by ultrasonography and CAP), and increased fibrosis markers (FIB-4, LSM, FAST score). Significantly, central obesity alone increased the probability of advanced fibrosis 3-fold after controlling for BMI and metabolic variables, a result that remained consistent in the MASLD subgroup, indicating that central obesity correlates with greater liver disease severity in lean NAFLD/MASLD patients.

This condition has given rise to the idea of ‘metabolically obese normal weight’ individuals (47). These people, while seemingly lean, are deemed metabolically unwell owing to latent metabolic dysfunctions, including aspects of insulin resistance, especially within adipose tissue, and compromised fat storage mechanisms (44). This signifies a crucial paradigm shift, demonstrating that BMI alone is an inadequate indicator of metabolic health and liver risk. Visceral adiposity, even without overall obesity, significantly contributes to the etiology of NAFLD. This emphasizes that fat distribution and the metabolic quality of adipose tissue are more important than total fat mass in assessing liver disease risk in lean subjects. Therefore, diagnostic screening for lean NAFLD should include more than basic BMI assessments and incorporate evaluations of central adiposity, such as waist circumference, to identify at-risk people (28).

#### 3.4.2 Insulin resistance and metabolic syndrome

Lean NAFLD patients often have metabolic risk markers such as dyslipidemia, hypertension, insulin resistance, and type 2 diabetes mellitus (11). Although these problems are detected less often or exhibit lower severity compared to their overweight or obese counterparts with NAFLD (11), they remain much more common than in healthy lean controls (13). Insulin resistance is a primary pathogenic mechanism for NAFLD, regardless of body composition (2). It enhances lipolysis in adipose tissue, resulting in the release of free fatty acids (FFAs) that are subsequently absorbed by the liver. This further facilitates *de novo* lipogenesis in the liver, whereby surplus glucose is transformed into fat (2). The persistent occurrence of insulin resistance and metabolic syndrome elements in lean NAFLD, however, is less severe than in obese individuals, indicating a threshold impact for metabolic dysfunction rather than a direct association with BMI. In fact, even minor metabolic disturbances in lean individuals might be enough to initiate and advance NAFLD development. This comprehension highlights the need for early identification and prevention of these ‘subclinical’ metabolic risk variables, since they are significant contributors to liver disease despite an apparently healthy body weight.

#### 3.4.3 Dietary habits and lifestyle factors

Dietary practices and lifestyle factors significantly influence the onset of lean NAFLD, often exhibiting distinct characteristics in contrast to the obese NAFLD phenotype. High fructose consumption, along with low protein and poor dietary fiber, is often linked to lean NAFLD (48). Excessive intake of foods rich in starch and carbohydrates may increase the risk of lean NAFLD (49). Significantly, elevated consumption of a fatty diet, even in the absence of excessive overall calorie intake, has been shown to lead to NAFLD in individuals of normal weight (44). This indicates that the quality and mix of the food, rather than only the caloric amount, are especially crucial in this phenotype. Lifestyle variables, including sedentary behavior, insufficient physical activity, and inadequate sleep patterns, are independently correlated with lean NAFLD (13). Indeed, research indicates that lean NAFLD patients often have reduced average weekly activity and daily sleep duration relative to healthy lean individuals (50). Collectively, this suggests that dietary strategies for lean NAFLD should be more specialized, concentrating on certain macronutrient limitations (e.g., sugar and cholesterol) instead of the broad caloric restrictions that is often recommended for obese persons. This customized strategy may be more effective due to the unique metabolic sensitivity of lean individuals.

#### 3.4.4 Genetic susceptibility

Genetic variables are becoming acknowledged as key contributors to the development of lean NAFLD, often exerting a more pronounced influence on disease onset and progression, sometimes independent of obesity or substantial insulin resistance. Key genetic markers implicated in lean NAFLD is the PNPLA3 (Patatin-like phospholipase domain-containing 3) rs738409 (I148M mutation) genotype. This variation is significantly linked to hepatic fat accumulation, inflammation, and more severe manifestations of NAFLD, such as NASH, fibrosis, and HCC (51). The prevalence is much greater in non-obese individuals than in obese patients with NAFLD (26). This mutation facilitates intracellular lipid buildup by diminishing the lipidation of very-low-density lipoprotein (VLDL) (26). The PNPLA3 rs738409 G allele is distinctly linked to elevated liver-related mortality and a heightened risk of NAFLD and severe fibrosis in lean individuals, often irrespective of conventional metabolic disorders (52). Another predominant genetic variant TM6SF2 (Transmembrane 6 superfamily member 2) rs58542926 (E167K variant), inhibits VLDL secretion from the liver, resulting in lipid buildup within hepatocytes and heightened vulnerability to liver injury and fibrosis, especially in lean individuals (53). The MBOAT7 (membrane bound O-acyltransferase domain-containing 7) genetic variant influence lipid droplet dynamics, resulting in diminished triglyceride secretion and heightened vulnerability to steatohepatitis and fibrosis (54). Other notable genetic variants include ApoB, LIPA, HSD17B13, GCKR, SIRT1, APOC3, AGTR1, PPARGC1A, CETP, SREBP, PEMT, and IFNL3/4, also have a role in disease susceptibility by affecting lipid metabolism, insulin resistance, and inflammatory pathways (55). The significant and occasionally autonomous influence of particular genetic variants in the pathogenesis of lean NAFLD indicates that, for a subset of lean individuals, the accumulation of liver fat and inflammation is predominantly caused by intrinsic hepatic lipid processing deficiencies rather than systemic metabolic overload due to generalized adiposity (56). This suggests that genetic screening might serve as an important method for detecting at-risk lean individuals, particularly in cultures with a significant incidence of these variations (e.g., certain Asian and Hispanic ethnicities). Thus, future treatment regimens could be directed toward gene-specific strategies, focusing on these unique biological pathways associated with hepatocellular lipid metabolism.

#### 3.4.5 Sarcopenia and muscle mass

Lean NAFLD is often linked to sarcopenia, defined by the gradual decline in both the amount and quality of skeletal muscle (13). This association is not accidental. A bidirectional relationship occurs in which sarcopenia may cause lean NAFLD, while the loss of muscle mass exacerbates ectopic fat deposition, especially in the liver. The incidence of sarcopenia is markedly elevated in individuals with NASH, underscoring its significance along the process of fatty liver disease (13). Sarcopenia is a significant risk factor associated with extensive hepatic fibrosis and elevated death rates (57). Sarcopenia may hinder glucose metabolism, since muscle serves as a principal location for insulin-mediated glucose elimination (10). Moreover, persistent inflammation and oxidative stress, often linked to sarcopenia, may adversely affect skeletal muscle and exacerbate liver damage (58). This complex interaction indicates that muscle health is not only a comorbidity but a direct factor in liver illness. Thus, therapies aimed at enhancing muscle mass and function, including resistance training and sufficient protein consumption, may provide a new therapeutic approach for lean NAFLD/NASH, either alone or in conjunction with conventional weight-loss methods.

#### 3.4.6 Differential metabolite profiles

Lean NAFLD patients often have distinctive metabolomic profiles in contrast to obese NAFLD patients or healthy lean people, offering insights into their specific metabolic dysregulations (59). Although they may exhibit elevated high-density lipoprotein (HDL) levels and reduced serum triglyceride and alanine transaminase levels relative to obese patients, a comparison with healthy lean controls indicates a contrasting scenario. Lean NAFLD patients have higher rates of hyperlipidemia, increased waist circumference, heightened serum triglycerides, low-density lipoprotein cholesterol, and blood glucose levels (60, 61). A significant change is seen in bile acid (BA) metabolism, with lean NAFLD patients exhibiting elevated levels of total BA, primary BA, and secondary BA in comparison to obese NAFLD patients (62). Alterations in BA composition, including reduced concentrations of deoxycholic acid, glycodeoxycholic acid, and goosenodeoxycholic acid, with elevated level of glycocholic acid have been documented (13, 63). Serum uric acid, an indicator of insulin resistance and the degree of liver impairment, is elevated in lean NAFLD patients compared to healthy controls, although HDL-c levels are diminished. The finding of these diverse metabolite profiles, especially the altered bile acid metabolism and specific lipid patterns, indicates that separate metabolic pathways are dysregulated in lean NAFLD compared to obese patients. This suggests that these divergent metabolites may function as possible non-invasive biomarkers for the early identification and monitoring of lean NAFLD, and might potentially signify new treatment targets for personalized therapies.

#### 3.4.7 Altered gut microbiota

Recent research suggests that individuals with lean NAFLD have a unique gut microbiota composition in contrast to obese NAFLD patients, underscoring the gut-liver axis as a potentially significant pathogenic mechanism in this phenotype. In the Indian lean NAFLD patients, an increased abundance of *Escherichia-Shigella* and depletion of *Faecalibacterium*, *Ruminococcus*, *Lactobacillus* and Lachnospira has been reported (64). In Chinese communities, individuals with lean NAFLD have reduced levels of Firmicutes (including Lachnospiraceae, Ruminococcaceae, Lactobacillaceae) and an elevation in lipopolysaccharide-producing Gram-negative bacteria (65). A comparable gut microbial profile highlighting a lower abundance of *Faecalibacterium*, *Ruminococcus* and *Lactobacillus* has been reported in lean NASH patients from a different cohort (66). Others reported that *Ruminococcaceae* and *Veillonellaceae* are the primary gut microbes linked to the severity of fibrosis in non-obese individuals (67). Caucasian individuals with lean NAFLD have an increased prevalence of Ruminococcaceae and *Dorea*, along with a reduction in *Marvinbryantia* and *Christensellenaceae* (13, 68).

Disruption of intestinal homeostasis, marked by heightened intestinal permeability and bacterial proliferation, facilitates the translocation of microbes and their metabolic byproducts (e.g., microbe-associated molecular patterns, MAMPs) from the gut to the liver, thereby exacerbating inflammation and injury (69, 70). This process is intensified by modifications in bile acid metabolism, which, in conjunction with abnormalities in the gut flora, may predispose people to the development of NAFLD even at lower BMI (13). The distinctive gut microbiome profiles in lean NAFLD indicate that dysbiosis and modified bacterial metabolites may be fundamental contributors to liver inflammation and fibrosis in this population. This creates opportunities for microbiome-targeted interventions, including dietary modulation, specific probiotic or prebiotic supplementation, and fecal microbiota transplantation, as innovative therapeutic strategies for lean NAFLD, potentially differentiating them from traditional methods primarily aimed at systemic metabolic regulation.

Collectively, this evidence clearly shows a distinct phenotype of lean NAFLD/NASH condition in terms of global burden, geographical variation, demographic heterogeneity and metabolic features (**Figure 1**).

## 4 Molecular pathogenesis: lean vs. obese fatty liver disease

**Figure 2:**
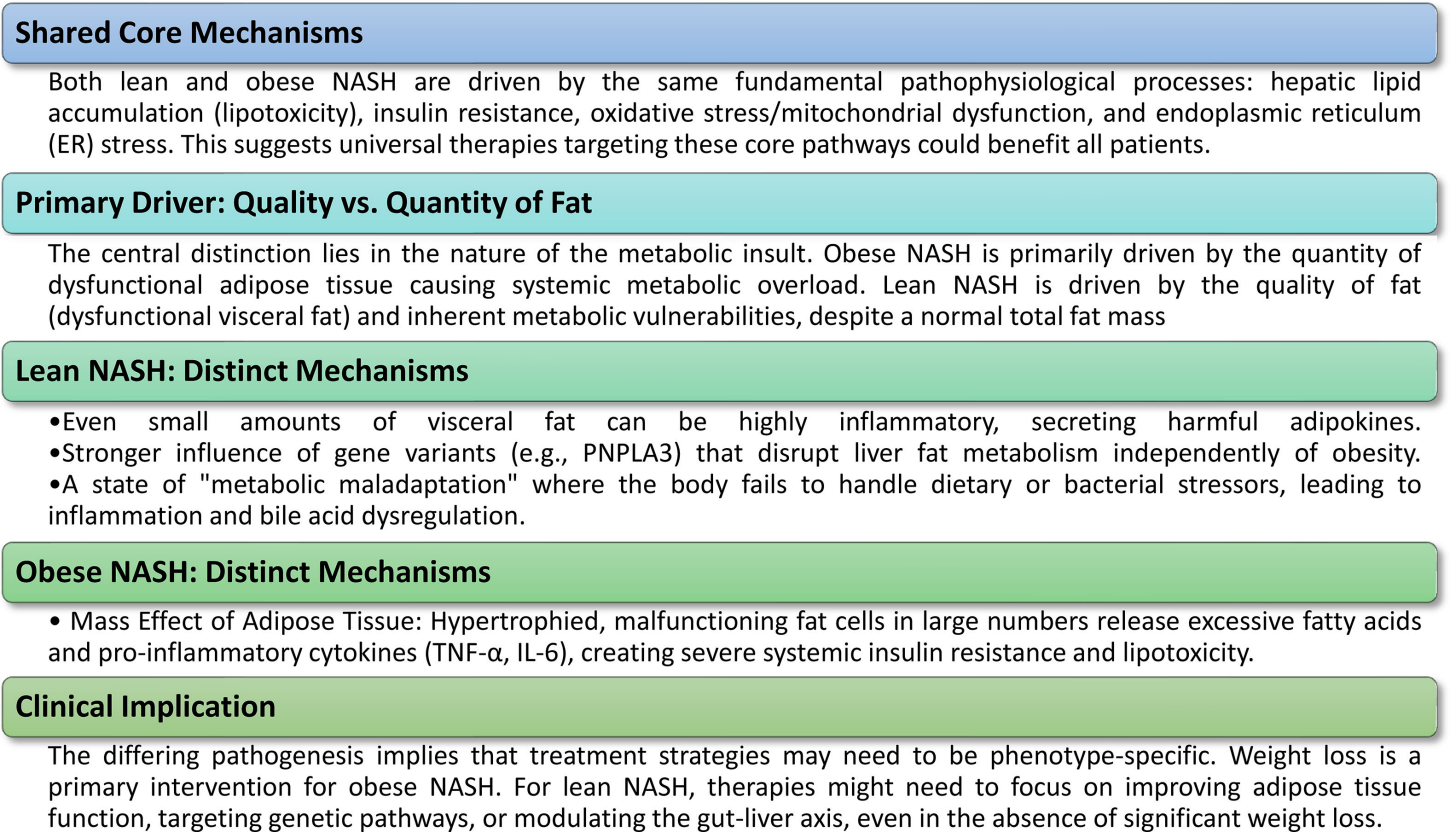
Molecular pathogenesis of lean vs. obese NASH. The molecular etiology of NAFLD/NASH includes both common core processes and unique, phenotype-specific pathways, elucidating why the disease can present similarly in persons with lean and obese BMI.

Comprehending the molecular etiology of NAFLD is essential for formulating targeted therapeutics. Although lean and obese phenotypes of NAFLD share core principles, separate pathways and contributing variables alter their underlying biology, providing insights into their diverse clinical presentations and prognoses (**Figure 2**).

### 4.1 Shared pathophysiological mechanisms

Regardless of variations in body composition, both lean and obese individuals with NAFLD/NASH are essentially influenced by analogous core cellular and molecular dysregulations. These similar processes provide the foundational basis upon which more distinct disease pathways develop.

#### 4.1.1 Hepatic lipid accumulation and lipotoxicity

The defining characteristic of both lean and obese NAFLD is the abnormal buildup of triglycerides in hepatocytes, referred to as steatosis (1). This buildup arises from a basic disparity between the energy source of the liver and its ability to use and eliminate it (2). The capacity of the liver to manage key metabolic energy sources, including glucose and fatty acids, gets exceeded (10). The origins of these hepatic fatty acids are diverse, including lipolysis from adipose tissue, *de novo* lipogenesis from surplus carbohydrates, and direct dietary lipid consumption (2). When the metabolic processes of the liver, including fatty acid β-oxidation and triglyceride export via very-low-density lipoprotein (VLDL) particles, become saturated, surplus FFAs are re-esterified and accumulated as triglycerides, resulting in hepatic steatosis (10). The buildup of these hazardous lipid species, termed ‘lipotoxicity,’ is a pivotal occurrence in the advancement from basic steatosis to NASH, instigating a series of cellular damage and inflammatory reactions (10).

#### 4.1.2 General insulin resistance

Insulin resistance is a crucial and common pathophysiological mechanism that underlies the development of both lean and obese NAFLD (2). In insulin resistance conditions, cells, especially those in adipose tissue, muscle, and liver, exhibit diminished responsiveness to insulin (71). This results in compensatory hyperinsulinemia, when the pancreas generates more insulin to counteract cellular resistance. At the cellular level, insulin resistance directly enhances *de novo* lipogenesis in the liver and indirectly by elevating the transport of FFAs to the liver owing to reduced suppression of lipolysis in adipose tissue (2). The subsequent accumulation of excess free fatty acids in the liver intensifies hepatic insulin resistance, establishing a detrimental loop (10). The existence of insulin resistance, irrespective of BMI, acts as a pivotal catalyst for the onset and advancement of NAFLD.

#### 4.1.3 Oxidative stress and mitochondrial dysfunction

Oxidative stress and mitochondrial dysfunction are fundamental elements in the development of both lean and obese NAFLD (72). The buildup of excessive lipids in hepatocytes enhances β-oxidation of fatty acids, potentially exceeding mitochondrial capacity and causing the production of reactive oxygen species (ROS) (10). The disparity between ROS generation and the hepatic antioxidant defenses induces oxidative stress, resulting in cellular damage to hepatocytes, lipids, and DNA (73). Mitochondrial dysfunction is a primary catalyst for hepatic and extrahepatic injury in NAFLD (74). Compromised mitochondrial activity may result in suboptimal energy production, increased ROS formation, and the activation of inflammatory and fibrogenic pathways (75). This common mechanism underscores that cellular energy metabolism and redox equilibrium are essential factors influencing disease development across the NAFLD spectrum.

#### 4.1.4 Endoplasmic reticulum stress

Endoplasmic reticulum (ER) stress constitutes a common pathogenic mechanism in both lean and obese NAFLD (73). The endoplasmic reticulum is an essential organelle responsible for protein folding, lipid production, and calcium regulation. In NAFLD, factors such as excess dietary energy, lipotoxicity due to lipid buildup, and oxidative stress may result in the aggregation of misfolded proteins inside the ER lumen (76). This activates the unfolded protein response, an adaptive process designed to restore endoplasmic reticulum equilibrium (76). Chronic or severe endoplasmic reticulum stress may become maladaptive, resulting in prolonged activation of the unfolded protein response that fosters inflammation, apoptosis of hepatocytes, and further impairs insulin signaling (73). Chronic ER stress strongly contributes to liver damage and fibrosis, serving as a consistent mechanism for disease development in both lean and obese patients with NAFLD. The recognition of these same core pathogenic processes highlights that, despite phenotypic variations, lean and obese NAFLD/NASH are essentially influenced by analogous cellular and molecular dysregulations. This suggests that universal therapy options aimed at these fundamental pathways, such as enhancing insulin sensitivity or diminishing lipotoxicity, may provide advantages throughout the whole range of NAFLD, irrespective of BMI.

### 4.2 Distinct molecular mechanisms in lean NASH

Although common pathways underpin the condition, the molecular pathogenesis of lean NASH is defined by unique factors that distinguish it from the obese counterpart. These distinct routes often explain why people with a normal BMI may nonetheless have severe liver disease.

#### 4.2.1 Adipose tissue dysfunction and ectopic fat deposition

In lean NAFLD, despite reduced total adiposity, there exists a specific pattern of fat distribution marked by increased visceral adiposity and ectopic fat accumulation in the liver (44, 77). This issue pertains not to the amount of fat, but to its metabolic nature. Dysfunction of adipose tissue results in a modified secretion profile of adipokines and pro-inflammatory cytokines, including TNF-α, IL-6, MCP-1, and CRP (78). Simultaneously, there is a decrease in anti-inflammatory mediators such as adiponectin (79). This imbalance fosters metabolic inflammation and leads to a persistent inflammatory condition in several tissues, including muscle. The notion of ‘dysfunctional adipose tissue’ in lean persons is a significant insight, indicating that the quality of fat, rather than only its amount, is a crucial factor in the pathogenesis of NAFLD in this population. This indicates that even a little amount of metabolically unfavorable visceral fat may initiate systemic inflammation and liver damage, differentiating it from the often larger but perhaps less dysfunctional adipose tissue seen in some obese individuals. This comprehension redirects attention beyond mere weight loss to enhancing the health and functionality of adipose tissue, even without substantial weight reduction.

#### 4.2.2 Altered adaptive metabolic responses

Lean NAFLD patients may have a modification in their adaptive metabolic response, marked by heightened blood bile acid levels and augmented farnesoid X receptor activation (40, 80). This indicates a deficiency in the defense systems to manage metabolic stress, even with reduced total adiposity. Proposed models of metabolic maladaptation loss aim to elucidate this phenomenon. The Western diet, characterized by elevated levels of certain dietary components, may modify intestinal permeability, resulting in heightened exposure to bacterial products, including lipopolysaccharides (40). In lean patients with NAFLD, this may lead to elevated endotoxemia, enhanced expression of macrophage TLR4, and increased production of inflammatory cytokines relative to healthy lean individuals (40). The notion of altered adaptive metabolic responses and metabolic maladaptation loss in lean NAFLD indicates a fundamental physiological susceptibility. This suggests that the condition in lean individuals may stem from an intrinsic metabolic vulnerability or a diminished ability to adjust adequately to external stressors, such as nutritional problems. This renders them vulnerable to hepatic damage despite an otherwise healthy phenotype. This comprehension indicates a profound, systemic problem extending beyond the liver, implying that slim persons may possess a diminished buffer against metabolic disturbances, rendering them more vulnerable to NAFLD while experiencing less severe external stresses.

### 4.3 Contrasting molecular mechanisms in obese NASH

To enhance comparative clarity, it is crucial to emphasize the divergent molecular processes mostly identified in obese NASH. Although both lean and obese NASH are characterized by inflammation and insulin resistance, the predominant factor in obese NASH seems to be the substantial quantity of defective adipose tissue and systemic metabolic excess (44). In obese individuals, surplus adiposity is often allocated both subcutaneously and viscerally, resulting in an elevated BMI and a heightened risk of metabolic diseases. In obese patients, adipocytes often undergo hypertrophy and malfunction, resulting in modified production of adipokines such leptin and adiponectin (76). This prevalent malfunction of adipose tissue greatly contributes to systemic insulin resistance by fostering persistent low-grade inflammation and ectopic lipid buildup in non-adipose tissues (73). The augmented release of FFAs from these impaired adipose tissues, together with heightened hepatic absorption of FFAs, results in significant hepatic triglyceride buildup and worsens hepatic insulin resistance (76). In obese NASH, inflammation is mostly driven by pro-inflammatory cytokines such as TNF-α, IL-1β, and IL-6, which are markedly elevated due to generalized adipocyte dysfunction and extensive insulin resistance (13). This inflammatory environment adds to hepatic damage and fibrosis. This stands in stark contrast to lean NASH, where certain genetic predispositions, qualitative fat dysfunction (even in minimal amounts), and dysregulation of the gut-liver axis may prevail despite reduced total adiposity. This difference is essential for understanding why certain therapeutic strategies, such as weight-loss programs compared to gene-targeted or microbiome-modulating medicines, may be more efficacious for each phenotype.

## 5 Clinical course, disease progression, and long-term outcomes

**Figure 3:**
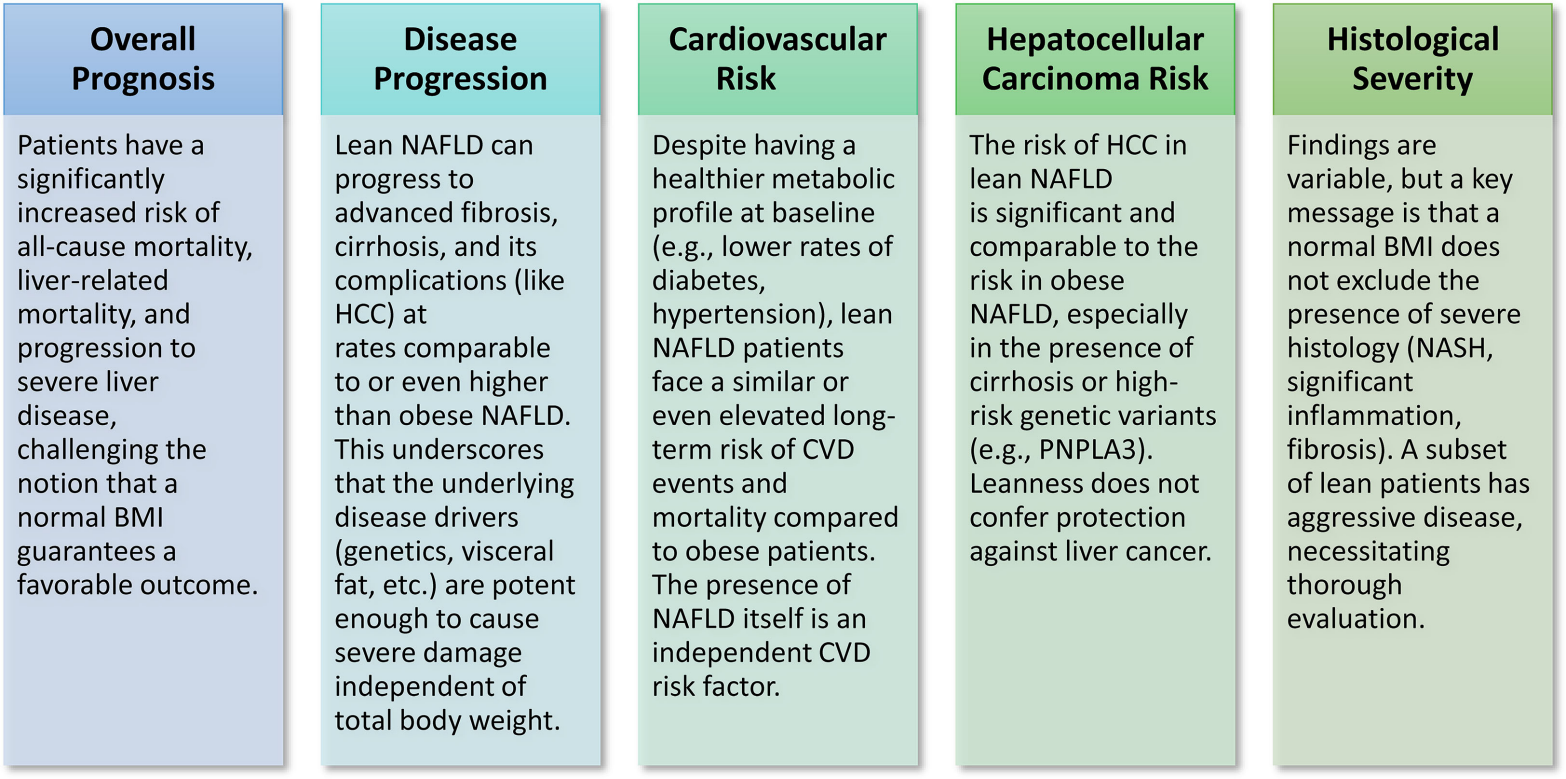
Clinical course & outcomes of lean NAFLD/NASH. Recent research challenges the traditional view that leanness offers protection, indicating that slim individuals with NAFLD/NASH face a considerable risk of serious liver and cardiovascular consequences, often comparable to or surpassing that of obese patients.

The clinical trajectory and long-term consequences of lean NAFLD/NASH are under investigation, with new findings disputing the previously accepted notion that leanness offers a safeguard against severe metabolic liver disease and extrahepatic consequences (**Figure 3**).

### 5.1 Histological features and disease severity

Individuals with a normal body weight who have NAFLD might have the characteristic histopathological signs of NASH, such as steatosis, lobular inflammation, and hepatocyte ballooning (21). This discovery is crucial, since it verifies that a normal BMI does not exclude the onset of substantial liver damage typical of NASH. Nonetheless, the histological severity of lean NAFLD in comparison to obese NAFLD remains a topic of controversy. Certain studies indicate that individuals with lean NAFLD may have very aggressive histological features, marked by heightened lobular inflammation and hepatocellular ballooning, hence increasing the risk of fibrosis and its advancement (44, 81). This viewpoint suggests that the fundamental pathogenic pathways in lean individuals are sufficiently robust to induce significant histological alterations. In contrast, several studies indicate that lean NAFLD patients are less prone to NASH or advanced histological changes and may have less hepatic steatosis compared to overweight or obese subjects (39, 44). Certain studies indicate decreased NAFLD activity scores and reduced hepatic fibrosis at presentation in lean individuals (26). The contradictory evidence on histological severity indicates considerable variability within the lean NASH phenotype. This diversity may be ascribed to disparities in the patient cohorts examined, the diagnostic techniques used, or the various underlying pathogenic factors (e.g., genetic vs nutritional impacts) within the lean NAFLD population. This indicates that a low BMI does not ensure a favorable histology outcome, and a portion of lean individuals may possess aggressive illness, require meticulous histological assessment when clinically warranted to appropriately evaluate disease severity and inform therapy.

### 5.2 All-cause and liver-related mortality

Recent meta-analyses indicate that people with lean NAFLD have an approximately 1.6-fold increased risk of all-cause death relative to those with non-lean NAFLD (82). This risk has been shown to be independent of age, sex, and conventional cardiometabolic risk factors (82). Although prior research proposed that lean NASH might be less severe or that patients would have a more favorable clinical trajectory (11), current investigations have contradicted earlier perspective. Some evidence suggests that lean NASH may have higher all-cause mortality and more advanced histological disease than obese NASH (13). Other studies, whilst reporting increased mortality in lean groups, have ascribed this to variations in age-related mortality, revealing no independent correlation with total mortality after age adjustment (83). Some studies have shown a substantial correlation between liver-related mortality and lean NAFLD, with one study showing an adjusted hazard ratio of 2.77 in comparison to obese NAFLD (82). This discovery is notably concerning and dispels the belief that lean NAFLD is harmless, emphasizing that metabolic health is not only indicated by BMI. Lean NAFLD patients need equivalent clinical attention and risk stratification as their overweight or obese counterparts, hence challenging existing screening and therapy paradigms that may otherwise neglect them.

### 5.3 Progression to advanced fibrosis and cirrhosis

NAFLD is a degenerative condition that may evolve from simple steatosis to NASH, and eventually to substantial fibrosis and cirrhosis (2). The existence of type 2 diabetes markedly elevates the risk of development to fibrosis and cirrhosis (17). Although some studies initially indicated that lean NAFLD patients may exhibit a lower baseline prevalence of advanced fibrosis compared to obese patients (26), an increasing body of evidence suggests that lean NAFLD can progress to advanced fibrosis and cirrhosis at rates comparable to, or even exceeding, those of obese NAFLD (14). Certain studies indicate an elevated risk of liver fibrosis advancement in individuals with lean NAFLD (76). This discovery undermines the idea that leanness provides a safeguard against terminal liver disease. A retrospective cohort study in Sweden indicated that lean NAFLD patients faced an elevated risk of developing cirrhosis, decompensated liver disease, or HCC, resulting in a higher incidence of liver-related mortality, despite a lower baseline prevalence of advanced fibrosis and steatohepatitis (82). A Chinese cohort research showed similar results, associating lean NAFLD with an elevated risk of liver-related death (82). The data indicated that lean NAFLD may develop to severe fibrosis and cirrhosis at rates comparable to obese NAFLD, although perhaps having fewer metabolic comorbidities, underscoring that leanness does not provide protection against catastrophic liver disease. This indicates that the fundamental pathogenic mechanisms in lean individuals, including particular genetic variants (e.g., PNPLA3 GG genotype), which influences NAFLD progression risk (82) and impaired adipose tissue, are sufficiently robust to promote fibrogenesis, regardless of total body fat. This emphasizes the essential need for fibrosis risk assessment in all NAFLD patients, irrespective of BMI, to guarantee prompt management and surveillance.

### 5.4 Hepatocellular carcinoma risk

NASH is a considerable risk factor for HCC, which has been shown in NASH patients without cirrhosis, however the risk is markedly elevated in those with cirrhosis (21). Specific genetic variations, including those in PNPLA3, TM6SF2, and MBOAT7, linked to lean NAFLD, are also associated with an elevated risk of HCC (73). Obesity is an independent risk factor for HCC (73); however, recent evidence indicates that lean NAFLD patients have a similar risk of HCC as obese individuals. Certain studies suggest that lean NAFLD patients possess a comparable risk of non-liver malignancies (84) and may exhibit an elevated risk of liver-related mortality, including HCC (82). A research including patients with type 2 diabetes revealed a markedly greater incidence of cancer among those with NAFLD, including non-obese or lean individuals, in comparison to those without NAFLD, with this correlation being regardless of BMI classifications (84). Emerging data suggests that the risk of HCC in individuals with lean NAFLD/NASH is equivalent to that in obese individuals, particularly in the presence of certain genetic predispositions, indicating that the carcinogenic potential of NASH is not exclusively linked to the level of obesity. This comprehension has significant ramifications for HCC monitoring protocols, indicating that risk classification for HCC should mostly emphasize the existence and severity of NASH and fibrosis, rather than depending on BMI as a principal criterion. Consequently, lean individuals diagnosed with NAFLD and exhibiting clinical indicators consistent with hepatic cirrhosis should get routine screening for HCC (16).

### 5.5 Cardiovascular disease risk

Cardiovascular disease (CVD) is the primary cause of death in people with NAFLD, a risk that persists irrespective of the BMI of the individual (71). This underscores that the existence of hepatic fat and inflammation is a significant independent risk factor for cardiovascular problems. Studies indicate that lean NAFLD patients often exhibit a markedly elevated atherosclerotic cardiovascular disease (ASCVD) score and a greater incidence of high ASCVD risk in comparison to obese NAFLD patients (85). This increased risk was determined to be independent of age, sex, and conventional cardiometabolic risk variables several investigations (82). Although lean NAFLD patients exhibit healthier cardiometabolic profiles at baseline - characterized by reduced diabetes prevalence, lower fasting blood glucose, smaller waist circumference, and decreased blood pressure - they may still encounter a comparable risk of CVD-related morbidity and mortality during follow-up when juxtaposed with non-lean NAFLD patients (28). The discovery that lean NAFLD presents a comparable or even elevated CVD risk relative to obese NAFLD, despite an apparent better baseline metabolic profile, is a significant and concerning revelation. The presence of hepatic steatosis and inflammation, regardless of BMI, is a significant independent risk factor for cardiovascular disease. This indicates that cardiovascular risk evaluation and therapy must be vigorously undertaken for all NAFLD patients, even those of normal weight, rather than relying only on conventional cardiometabolic risk factors or BMI. Clinicians must acknowledge that a normal weight does not eliminate the substantial cardiovascular risk associated with underlying fatty liver disease.

## 6 Management and surveillance considerations for lean NASH

The care of lean NASH, while aligned with the core concepts of obese NASH, requires particular modifications owing to the unique clinical and molecular characteristics of this patient group.

### 6.1 Lifestyle interventions: diet and exercise adaptations

Lifestyle intervention, including dietary changes and enhanced physical activity, is fundamental NAFLD/NASH therapy for all patients, regardless of body weight (71). This universal recommendation highlights the essential function of energy balance and metabolic health in liver disease. A crucial adaption for lean NAFLD patients is the objective of weight reduction. Obese individuals generally need to lose 7-10% of their body weight to realize substantial enhancements in hepatic steatosis and NASH remission, whereas lean patients can attain comparable benefits, such as notable reductions in steatosis and potential NAFLD remission, with a more modest weight loss of 3-5% (44). The reduced target for weight loss is a significant practical consideration, indicating that management strategies for lean individuals should prioritize achieving these more feasible, modest reductions and specific dietary modifications instead of pursuing aggressive weight loss, which may be less suitable or sustainable for them. Particular dietary modifications are crucial for lean NAFLD. It is particularly recommended to restrict the use of fructose and sugar-sweetened beverages, especially for younger, lean individuals, owing to their significant correlation with the disease and their involvement in *de novo* lipogenesis (44). A concentration on a low-fat diet may be more suitable for lean individuals with NAFLD (86). High-carbohydrate diets have been recognized as probable predispositional variables for lean individuals (44). Aerobic and anaerobic exercise are linked to a decrease in liver fat and provide extensive metabolic advantages, often irrespective of substantial weight loss (71). Resistance training may be particularly advantageous for lean individuals, especially those with sarcopenia, since it addresses muscle mass and quality, which are essential components in the etiology of lean NAFLD (86). These customized lifestyle treatments are crucial for addressing the specific metabolic sensitivity and body composition of lean persons with NAFLD.

### 6.2 Pharmacotherapy: current evidence and future directions

Despite the rising incidence and clinical importance of NASH, there are no FDA-approved therapies especially for its management, however many pharmaceuticals are in different phases of research (87). The reliance mostly persists on lifestyle changes. For biopsy-confirmed NASH, certain pharmacological treatments may be considered.

Vitamin E (800 IU daily) may be used for lean individuals with biopsy-confirmed NASH who lack type 2 diabetes or cirrhosis (16). Likewise, pioglitazone (30 mg/d), a thiazolidinedione, may be planned for lean individuals with biopsy-confirmed NASH, irrespective of their type 2 diabetes status, as long as they do not have cirrhosis (16). The use of both vitamin E and pioglitazone must be confined to individuals with biopsy-confirmed NASH to guarantee suitable patient selection (16). Glucagon-like peptide-1 (GLP-1) receptor agonists (e.g., liraglutide, semaglutide) and sodium-glucose cotransporter-2 (SGLT2) inhibitors are recognized for their effectiveness in controlling hyperglycemia and mitigating cardiovascular risk in individuals with type 2 diabetes (21). These pharmacological classes have also shown efficacy in enhancing hepatic steatosis, steatohepatitis, and mitigating fibrosis development in the wider NAFLD/NASH cohort (88, 89). Nonetheless, a notable deficiency in existing research is the lack of evidence about the therapeutic effectiveness of GLP-1 agonists and SGLT2 inhibitors, especially in the treatment of lean NAFLD (16). The majority of current clinical studies for these medications primarily include overweight and obese patients, resulting in a lack of knowledge about their unique advantages and recommended dose for lean people with different pathologic profiles. Therefore, while these drugs may be contemplated for the management of concomitant metabolic disorders such as type 2 diabetes in lean NAFLD patients, their direct use for thin NASH therapy is still premature for more targeted clinical studies for this underserved demographic (16).

### 6.3 HCC surveillance guidelines

HCC is a significant consequence of advanced liver disease, making its monitoring essential for early identification and improved outcomes. For individuals with lean NAFLD, biannual HCC monitoring using abdominal ultrasonography, perhaps augmented by serum α-fetoprotein testing, is recommended if clinical indicators imply liver cirrhosis (16). This suggestion is based on the recognized knowledge that cirrhosis, irrespective of its etiology, is a significant risk factor for HCC (16). The prevalence of HCC in non-cirrhotic NAFLD is markedly reduced, indicating that monitoring initiatives should mostly target individuals who have advanced to cirrhosis (16). Population-wide screening for NAFLD is still not advised (16). Screening for NAFLD and severe fibrosis should be prioritized in high-risk groups, including those with type 2 diabetes or metabolic syndrome (90). The guidance for HCC surveillance in lean NAFLD patients with cirrhosis emphasizes that advanced liver disease, rather than BMI, is the principal factor influencing cancer risk and monitoring. This indicates that clinicians must actively assess lean NAFLD patients for fibrosis and cirrhosis, since their lean physique does not exempt them from the risk of acquiring end-stage liver disease and its consequences, including hepatocellular carcinoma. This proactive strategy guarantees that even people with apparently healthy weights undergo suitable surveillance for this life-threatening condition.

## 7 Conclusion and future prospects

**Figure 4:**
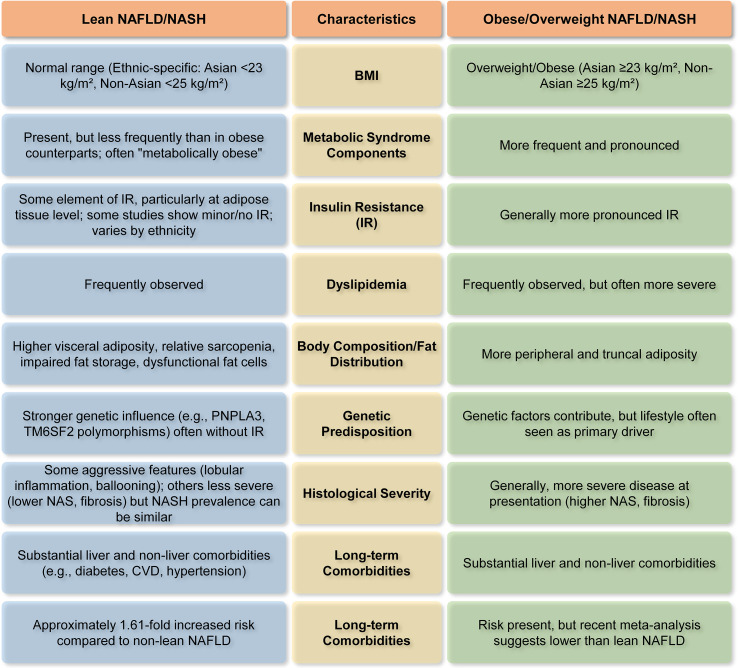
Major differentiating characteristics of lean vs. obese NAFLD/NASH (14, 26, 32, 44, 82). This figure highlights how NAFLD/NASH patients with or without obesity differ in terms of BMI, metabolic parameters, body composition, genetic predisposition, etc.

The emergence of lean individuals with MASLD, is a significant and developing problem in hepatology. Indeed, there is a clear distinction between NAFLD/NASH patients with or without obesity (**Figure 4**). This thorough study emphasizes that lean NAFLD may not be a trivial disease but a distinct and substantial public health issue. Epidemiological studies validate its significant and rising worldwide frequency, exhibiting considerable differences across geographical locations and ethnic groupings, especially a greater burden in Asian populations. Despite increasing recognition, substantial deficiencies persist in the validation of lean NAFLD. A significant problem is the absence of a defined, widely recognized definition, especially for BMI cut-offs, which differ by ethnicity and study, so complicating epidemiological comparisons and risk evaluations (11). Moreover, current NITs and biomarkers have predominantly been formulated and validated within obese cohorts, resulting in restricted accuracy and dependability for identifying and staging disease in lean individuals (16). A significant deficiency exists in validated, lean-specific non-invasive biomarkers for early diagnosis and fibrosis assessment. The evidence supporting pharmacotherapy is inadequate, as the majority of clinical trials using drugs such as GLP-1 agonists and SGLT2 inhibitors have primarily included obese individuals, resulting in the efficacy and safety of these treatments for lean NAFLD remaining largely unverified (16). Rectifying these validation deficiencies is crucial for enhancing diagnosis and formulating focused management strategies for this specific phenotype.

A key finding is that, although possessing a normal BMI, lean NAFLD patients may have underlying metabolic irregularities, often marked by elevated visceral adiposity, resulting in a ‘metabolically obese normal weight’ phenotype. This questions the conventional dependence on BMI as the only measure of metabolic health and liver risk. Although BMI serves as a useful first screening tool, it possesses considerable limitations in evaluating NAFLD risk, since it fails to distinguish between adipose and lean muscle mass or consider the distribution of fat (91). Visceral adipose tissue (VAT), especially the fat accumulated in the abdominal region, is a significant contributor to inflammation and insulin resistance in NAFLD, even among persons with a normal BMI (77, 92, 93). Consequently, including supplementary body composition measurements can yield a more precise risk evaluation. Consequently, augmenting BMI with metrics such as waist-to-hip ratio, VFA, or sophisticated body composition imaging could provide a more effective method for identifying persons at risk, even those with normal-weight NAFLD, by emphasizing pathogenic visceral adiposity rather than solely overall weight (77). Moreover, the molecular pathogenesis of lean NASH, although it shares fundamental mechanisms with obese NASH, is characterized by significant genetic predispositions (e.g., PNPLA3, TM6SF2, MBOAT7), dysfunctional adipose tissue (even in lesser amounts), distinct gut microbiome signatures, and modified adaptive metabolic responses. These unique factors indicate that inherent hepatic lipid processing deficiencies and certain environmental interactions are more influential in lean individuals. Importantly, lean NAFLD is linked to similar, and in certain analyses, even elevated risks for severe long-term outcomes, such as all-cause mortality, progression to advanced fibrosis and cirrhosis, hepatocellular carcinoma, and cardiovascular disease, in comparison to overweight or obese individuals. This substantially contradicts the misconception that leanness provides protection against severe hepatic and extrahepatic metabolic dysfunctions.

Despite considerable progress, some essential concerns remain, requiring targeted research goals to enhance the diagnosis and therapy of lean NAFLD/NASH. For instance, a generally recognized and consistent definition of lean NAFLD/NASH, with specific BMI thresholds and a thorough evaluation of body composition beyond BMI, is essential. This standardization will significantly boost epidemiological comparability across studies and improve the precision of research endeavors (11). Additional research is required to thoroughly clarify the specific molecular pathways exclusive to lean NAFLD/NASH. This involves elucidating the complex interactions among certain genetic predispositions, the qualitative impairment of adipose tissue (even in little amounts), and the unique modifications in the gut flora. Comprehending these intricate relationships will provide a more profound insight into disease onset and advancement in this phenotype (13). The advancement and validation of more precise, accessible, and sensitive non-invasive biomarkers and imaging methodologies are essential. These instruments are crucial for early diagnosis, accurate fibrosis staging, and monitoring of lean NAFLD/NASH, particularly because of the asymptomatic characteristics and under-recognition of the disease in this general population (11). Targeted clinical studies are critically required to assess the effectiveness and safety of pharmacological interventions, notably GLP-1 receptor agonists and SGLT2 inhibitors, primarily in lean NAFLD/NASH populations. Current studies are mostly composed of overweight and obese patients, resulting in a substantial information deficit about appropriate therapy approaches for lean individuals (16). Investigations into gene-targeted therapeutics, informed by recognized genetic predispositions, show considerable potential. Additional prospective, rigorously constructed longitudinal studies are necessary to comprehensively delineate the natural history, disease progression rates, and long-term effects of lean NAFLD/NASH in various populations. This will clarify discrepancies in histology severity and mortality risk, offering a more precise understanding of prognosis (44). Research must concentrate on formulating and validating comprehensive management strategies that amalgamate customized lifestyle interventions (e.g., specific dietary alterations beyond caloric restriction, targeted exercise for sarcopenia) with suitable pharmacological methods, taking into account the distinct metabolic and genetic profiles of lean individuals (16). The increasing acknowledgment of lean NAFLD as a unique and possibly severe condition requires a transformation in clinical practice and research. To advance, a cooperative, multidisciplinary strategy is crucial to resolve these unresolved inquiries, therefore enhancing diagnosis, risk assessment, and tailored care for these at-risk patient populations.
